# Construction Notes

**Published:** 1893-04-01

**Authors:** 


### CONSTRUCTION NOTES.

Small-pox having broken out in the Hemsworth district in Yorkshire, the rural sanitary authority have decided to erect a fever hospital. Mr. Wrightson (formerly of Cusworth Hall, Yorkshire), has generously offered £300 for this purpose. The building will be known as he Wrightson Fever Hospital.

